# Supplementary material for: The Roles and Regulatory Mechanisms of Tight Junction Protein Cingulin and Transcription Factor Forkhead Box Protein O1 in Human Lung Adenocarcinoma A549 Cells and Normal Lung Epithelial Cells
Source: Int J Mol Sci. 2024 Jan 24;25(3):1411. doi: 10.3390/ijms25031411 (PMC10855320; doi:10.3390/ijms25031411)
Supplement: Supplementary file 1 [file ijms-25-01411-s001.zip › ijms-2774493-supplementary.pdf]

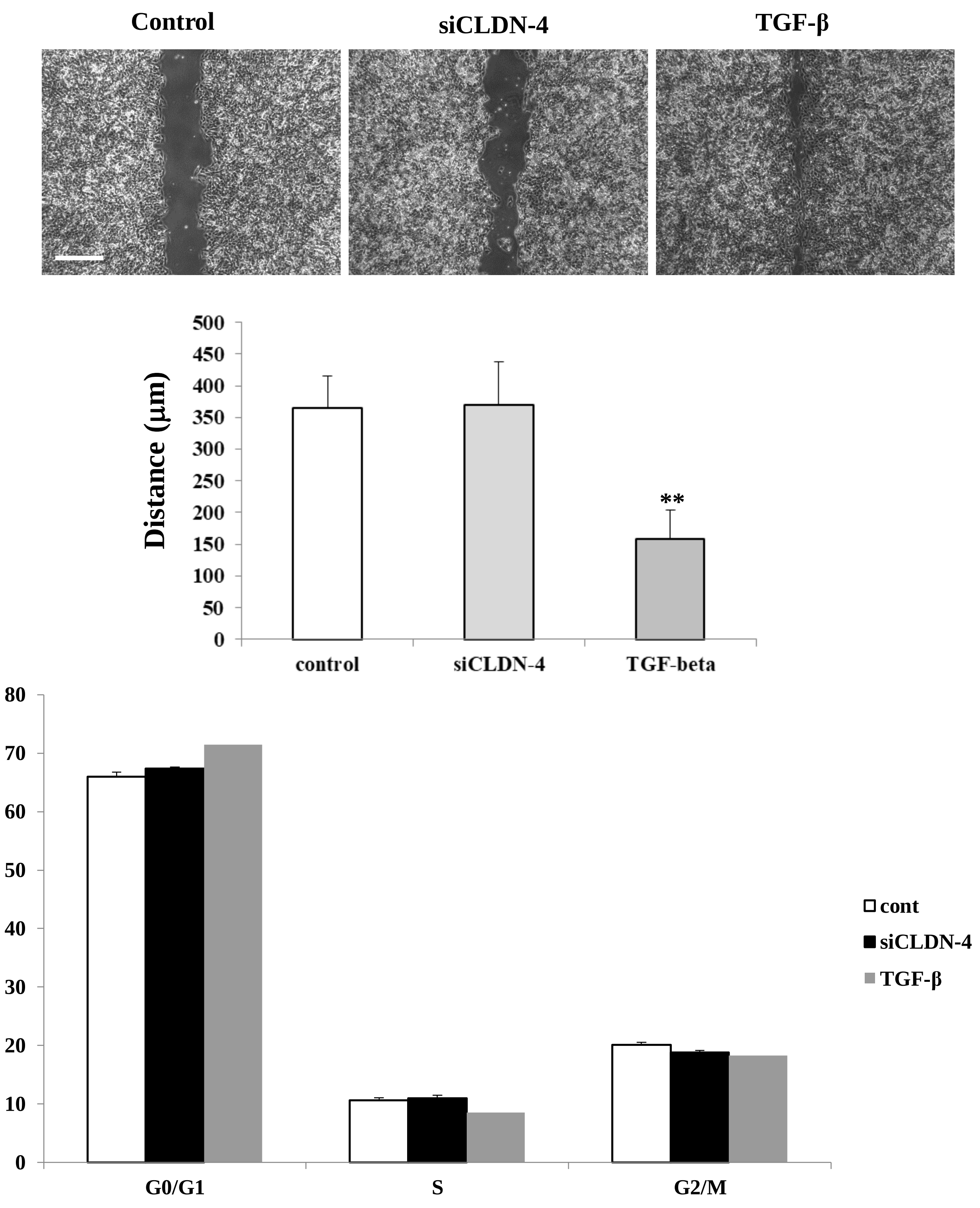

Supplemental Figure S1: Images of scratch wound assay in A549 cells performed knockdown of CLDN-4 and treated with TGF-β. The distance is shown as a bar graph. Scale bars: 200 μm. **p < 0.01, vs. control. Cell cycle assay of A549 cells performed knockdown of CLDN-4 and treated with TGF-β. The results are shown as bar graphs. ***p* < 0.01, vs. control.

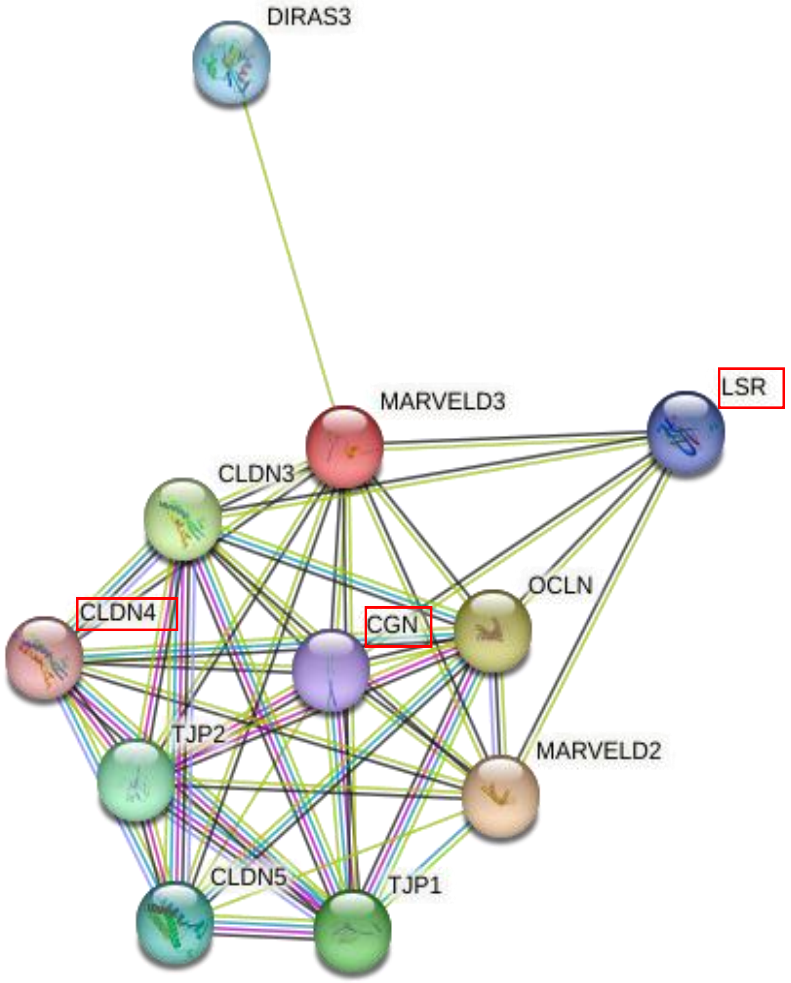

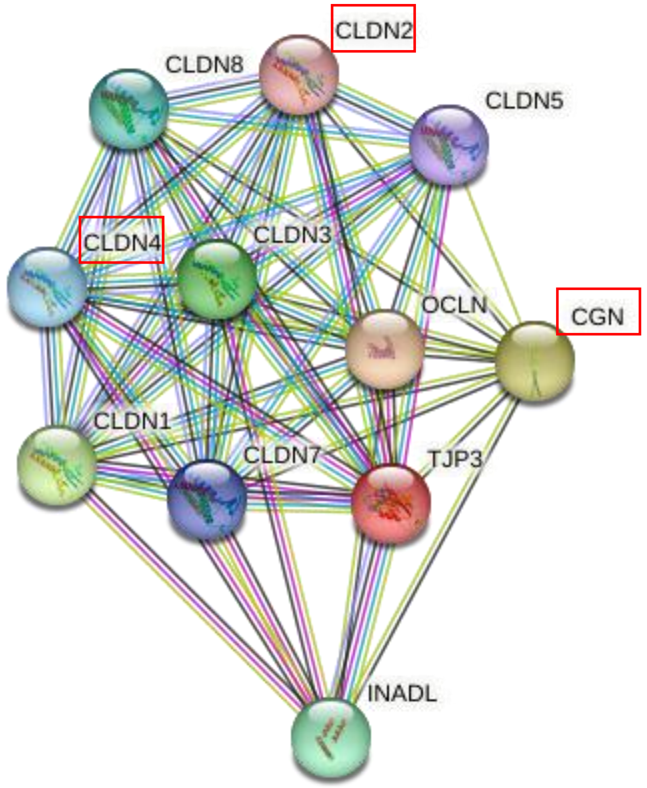

Supplemental Figure S2: Protein-protein interaction map. CGN directly interacts with CLDN-2, CLDN-4 and LSR.

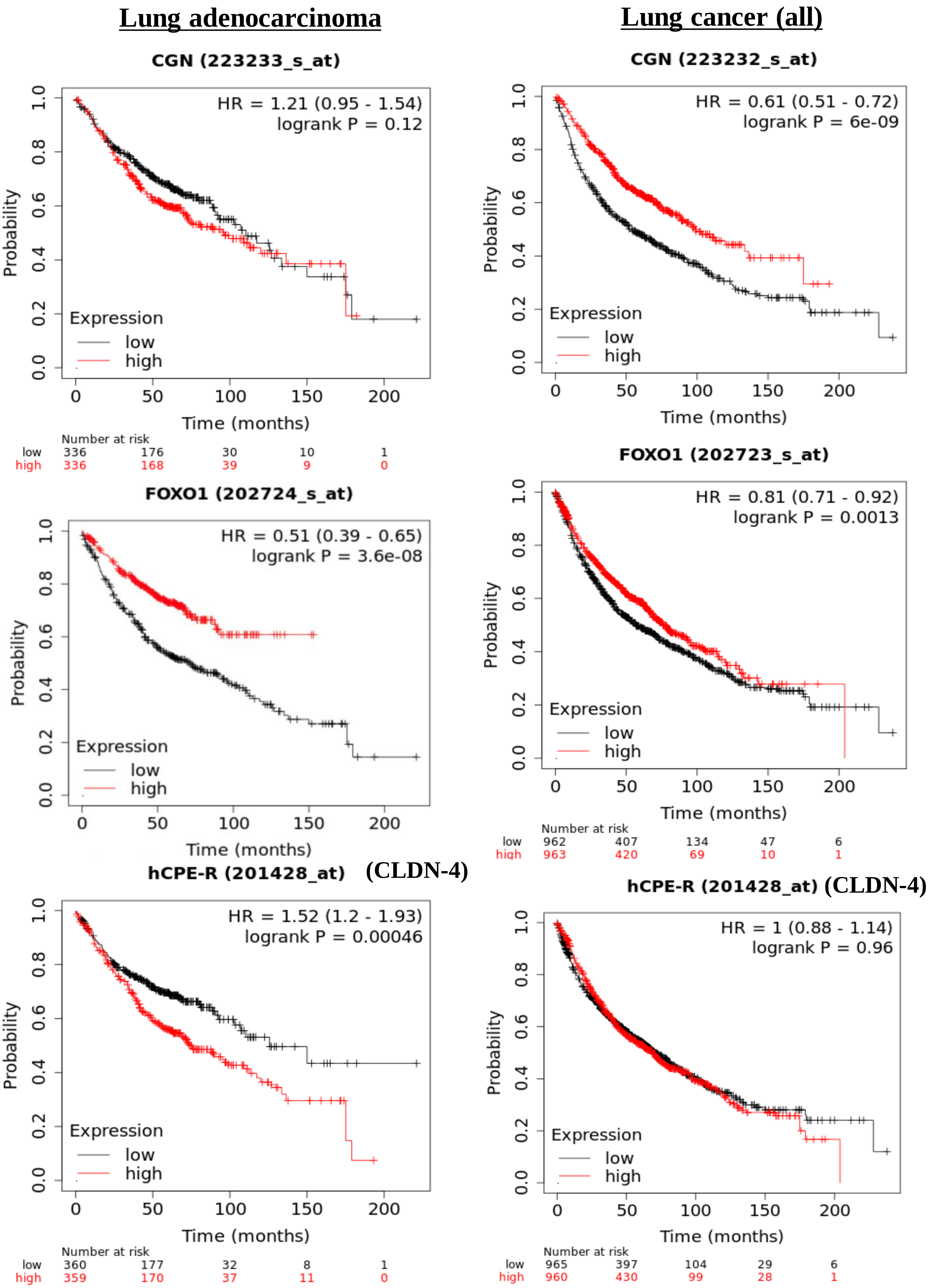

http://kmplot.com/analysis/index.php?p=service&cancer=lung

Supplemental Figure S3: TCGA shows the relation of CGN, FOXO1, and CLDN-4 expression and survival rate in lung cancer.
